# A Working Hypothesis for the Mode of Carcinogenesis of Aromatic Amines

**DOI:** 10.1038/bjc.1953.48

**Published:** 1953-12

**Authors:** D. B. Clayson


					
460

A WORKING HYPOTHESIS FOR THE MODE OF

CARCINOGENESIS OF AROMATIC AMINES.

D. B. CLAYSON

Department of Experimental Pathology and Cancer Research,

School of Medicine, Leeds, 2.

Received for publication August 17, 1953.

A CONSIDERABLE amount is known about the carcinogenic properties of
certain aromatic amines and azo-compounds. In this paper a working hypothesis
is put forward for the mode of carcinogenic action of these compounds and the
evidence to support it is discussed. A working hypothesis would be of value
not only as a basis for further systematic work in this field but ultimately if it
could be established as having validity in prediction it would be useful in selecting
any suspected compounds of industrial importance for full biological investiga-
tion. No attempt has been made in this account to review exhaustively the
literature of aromatic amine carcinogenesis: only those papers which have a
direct bearing on the argument will be quoted.

Those aromatic amines which are known to be carcinogenic can be regarded
as derivatives of aniline in which the para position is substituted by a large
conjugated group. (In the examples quoted the para position is marked by
an asterisk.) The amino group may carry certain substituents which the body
is capable of removing (Williams, 1947). Thus, in addition to the carcinogenic
aromatic amines themselves, derived acetamides, diacetylamines, and mono-
and dimethylamines have been shown to be carcinogenic. Similarly, some
corresponding nitro-compounds are active and it is known that the nitro group
may be reduced to an amine in vivo (Robinson, Smith and Williams, 1951).

The carcinogenic aromatic amines have been found to induce tumours in a
wide variety of sites in different species as is shown in the accompanying tables.
The relatively large doses of the aromatic amines required to induce tumours
has resulted in the use of feeding in preference to injection for their administration.
This may be the reason why carcinomas are more frequent than sarcomas. The
amino-stilbenes (Haddow, Harris, Kon and Roe, 1948), for example, gave rise to
sarcomas in both rats and mice on subcutaneous injection. There is a tendency
for aromatic amine carcinomas to occur in the liver; sites on the various routes
of excretion such as the bladder, ureters and intestine; and also, in the case of
the rat, the acoustic gland. The widespread distribution of tumours suggests
that the active agents circulate and induce tumours where their concentration
and other factors are favourable. In support of this Bielschowsky (1944) has
shown that stimulation of the thyroid of the rat by allyl thiourea results in the
formation of 2-acetamidofluorene tumours in this gland.

Different species respond differently to the same aromatic amine. 2-Naph-
thylamine (I), for example, induces tumours readily in the dog (Hueper and
Wolfe, 1937; Bonser, 1943) and with difficulty in the rat and rabbit (Bonser,

MODE OF CARCINOGENESIS OF AROMATIC AMINES

Clayson, Jull and Pyrah, 1952). Benzidine (II) tumours are induced readily in
the rat and with difficulty, if at all, in the dog (Spitz, Maguigan, and Dobriner,
1950). These differences in the tumour incidence in different species suggest
that (i) it is a metabolite rather than the amine itself which is responsible for the
carcinogenicity of the aromatic amines and (ii) that metabolism varies from
species to species (as has already been shown to be the case with some amines).

Bonser, Clayson and Jull (1951) have obtained evidence that 2-amino-l-
naphthol (III) (or one of its derivatives) is the carcinogen responsible for the
tumours induced by 2-naphthylamine in the dog, mouse, rat and rabbit. Re-
cently they (Bonser et al., 1952) have shown that whereas 2-amino-l-naphthol is
locally active, 2-naphthylamine is not. In its quantitative aspects, the con-
version of 2-naphthylamine to 2-amino-l-naphthol derivatives, offers an explana-
tion of the differences in species susceptibility to 2-naphthylamine and for the
localisation of tumours in the bladder of the dog, rat and rabbit, and in the
liver of the mouse. Baker (1951) has obtained some evidence to show that
(i) the urine of men exposed to benzidine contains a carcinogen and that (ii)
3: 3'-dihydroxy-benzidine (IV), a probable metabolite of benzidine, is carcino-
genic (Baker, 1952). He has published no evidence to show that this compound
is a local carcinogen. Walpole, Williams and Roberts (1952) quote a personal
communication from Baker that, not only were the intestinal tumours with
3: 3'-dihydroxy-benzidine obtained earlier than those with benzidine in the
parallel experiments of Spitz et al. (1950) and Walpole et al. (1952), but that he had
also obtained papillomas of the forestomach. No inference can safely be drawn
from the first observation, as the use of different strains and different means of
administration might make considerable differences to the time of appearance
of the tumours. The latter observation is of doubtful significance as Bonser
et al. (1952) found forestomach tumours in 30 per cent of control rats and of
those fed 2-naphthylamine in their food. Proof of the contention of Walpole
et al. (1952) that 3: 3'-dihydroxy-benzidine is indeed the effective metabolite
can only be found by the direct investigation of the local action of 3: 3'-dihy-
droxybenzidine on the tissues. Bielschowsky (1945) isolated 7-hydroxy-2-
acetamido-fluorene (V) from the acetamidofluorene (VI) urine of the rat. Hoch
Ligeti (1947) suggested that this metabolite was a weak carcinogen, but as in
her experiment the controls were killed at 500 days and most of the tumours in
the experimental group occurred between 500 and 700 days this observation
requires confirmation.

The present hypothesis is an attempt to bring these first results into orderly
relationship. It is suggested that:

(i) compounds which contain a hydroxyl and an amino group ortho to each
other in an aromatic system of two or more rings may be carcinogenic either in
their own right or as a result of their further reaction; (ii) the reason why some
aromatic amines induce tumours whereas others do not is that the former are
more readily converted in the body to ortho-hydroxy amines than the latter.

Hydroxylation, and acetylation, appear to be the main detoxication
mechanisms undergone by the aromatic amines. Aniline, for example, is con-
verted by the rabbit to derivatives of para- and to a lesser extent ortho-amino-
phenol (Smith and Williams, 1949). Williams (1947) in his excellent review
indicated that other aromatic amines behave similarly. It is now suggested
that those aromatic amines which have a blocked para position will tend to

461

D. B. CLAYSON

// \// \\\ NH2

11 *

(I)

OH

//\,// \\   NH2

1   1

(III)
CH2

HO     \\\      /

11    11
11    II

I \I
(V)

NHCOCH3

'X/

hydroxylate to a greater extent in the ortho position and will thus lead to the
postulated carcinogens, as has been shown to be the case with 2-naphthylamine.

NH2

//\  OH

l11 -

I1 <

NH2

11

NHi2

11

II

OH

The experimental testing of such a hypothesis requires three lines of approach:
(i) proof of the carcinogenicity of the ortho-hydroxy amines; (ii) demonstration
that the carcinogenic aromatic amines are, in fact, convxerted to ortho-hydroxy
amines in vivo; and (iii) demonstration that the carcinogenic metabolites, if
formed, reach the sites of election of the tumours induced by the original aromatic

x

H  *X H IO

x

-NH.,;

OH

X  *      NH2    >         --NH"

H2N/

___ W*

-NH2

(1I)

HO
H2NX

OH

/ -NH2

(IV-)

CHe

Ii

11*

NHCOCH3

(VI)

462

MODE OF CARCINOGENESIS OF AROMATIC AMIINES

amines. A start has been made in Leeds on the first of these problems in the
last eighteen months. A number of ortho-hydroxy amines are under test by the
bladder implant technique (Jull, 1951) in which a paraffin wax pellet containing
the chemical is introduced surgically into the mouse bladder. These substances
include: 1-amino-2-naphthol, 3-amino-4-hydroxy-biphenyl, 3-amino-2-naphthol,
I-amino-2-naphthol-4-sulphonic acid, 3: 3'-dihydroxybenzidine and ortho-amino-
phenol. A commercial sample of 1-amino-2-naphthol (VII) hydrocholride has
been found to induce tumours in five of six mice surviving for more than 16
weeks (Bonser and Jull, personal communication).

Detailed consideration of the aromatic amines:

It is difficult to subdivide the known carcinogenic aromatic amines into
further classes although they can be regarded as derivatives of 2-naphthylamine
(II), 4-aminobiphenyl (VIII) or 4-aminostilbene (IX). The evidence for the
nmode of action of 2-naphthylamine has already been considered. The first

OH

I                                                NI|\., <\- H.

(Vill)

__tH          'H--     ---7 H

(IN)

published study of the aminostilbenes (Haddow et al., 1948) was nmore concerned
with their tumiour inhibitory properties than with their carcinogenic action.
In view of the suggested connection between tumour inhibition and carcinogeni-
city it is interesting to notice that those stilbenes found to inhibit the growth of
the test tumour significantly were all para-substituted anilines: but it should
be noted that by no means all para-substituted anilines in this series were tumour
inhibiting. Thus it appears that factors other than para-substitution of an
amine are of importance.

By far the most extensively investigated is the 4-aminobiphenyl series which
includes compounds of the 2-aminofluorene type. With the exception of 2-
aminofluorene, however, most of the work has been carried out in the rat only.
4-Aminobiphenyl (VIII) (Walpole et al., 1952), its acetamide (Miller, Miller,
Sandin and Rusch, 1952) and 4-dimethylaminobiphenyl (Miller, Miller, Sandin
and Brown, 1949) have been shown to induce turnours in the rat. Various
methyl homologues have been found to be active including 3: 2'-dimethyl-,
(X), 3: 3'-dimethyl (XI) and 3-methyl-4-aminobiphenyl (XII). On the other
hand 2-methyl- (XIII) and 2'-methyl-4-acetamidobiphenyl (XIV) were found
not to induce tunmouirs in the rat. The methylene bridge in 2-amino-fluorene

463

D. B. CLAYSON

may be replaced by various other substituents (XV-XVIII) and a fluorine atom
may be substituted in the 7-position in this compound (XIX) without loss in
activity.

CH3

CH3

CH3

-N'H2

\NH2

(XI)

CH3

\NH2

\NHCOCH3

(XIV)

0
'I

S

CH3

\-N'HCOCH3

(XLII)

0

411'     X\-NICOCH3

\==,

(XV)

S

-7

NHCOCH3

-NHCOCH3

(XVI)
CH = C'H

/      \\  9     )SNHCOCH3

(XVIII)

(XVII)
CH2

FX                  I

(XIX)

Miller et al. (1952) explain their failure to induce tumours in the 2- and 2'-
methyl-4-acetamidobiphenyls by suggesting that the substituent destroys the
planarity of the molecule, and thus its carcinogenicity. This is clearly inadequate
as 2': 3-dimethyl-4-aminobiphenyl, which was found to be highly active by
Walpole et al. (1952), contains a methyl substituent in the 2'-position. The
contradiction might be explained by differing metabolism of the compounds.
As Walpole et al. (1952) have noted, o-acetotoluidine is hydroxylated whereas
m-acetotoluidine is oxidised to meta-acetamido-benzoic acid by the dog, and it
seems possible that the corresponding aminobiphenyls behave similarly in the rat.

CH3

(X)

(XII)

CH3

,-NHCOCH3

464

MODE OF CARCINO(GENESIS OF AROMATIC AMINES

Walpole et al. (1952) make a special point of the enhancing of the activity of
the 4-amninobiphenyl derivatives by the 3-methyl substituent. While their
view provides a satisfactory explanation of the known carcinogenic activitiesof
the methyl-4-aminobiphenyls, other substitutions can also be made without
destroying carcinogenic activity. For example, a methylene bridge in the

2: 2'- position as in 2-aminofluorene converts 4-aminobiphenyl into one of the
most ubiquitous and potent carcinogens known, and according to Miller et al.
(1952) substitution of the 7- position with fluorine in 2-acetamnidofluorene may
enhance this activity still further. If, as is now suggested, metabolisnm is a pre-
requisite to carcinogenic activity, substituents might have one or more of the
following effects : (i) a substituent might alter the reactivity of the molecule
and render it more or less easily metabolised  (ii) a substituent might act as an
additional centre for metabolism, as in the oxidation of a methyl to a carboxyl
group, and thus reduce the amount of the carcinogenic metabolite formed;
(iii) a substituent might block one of the routes of metabolism of a compound
and thus alter the formation of a carcinogenic metabolite; (iv) a substituent
might affect the ease of interaction of a carcinogenic -metabolite with the tissue.
As only a score of aromatic amines have been adequately tested for carcinogeni-
city it is not surprising that it is still impossible to predict the effects produced
by any particular substituent. Nevertheless, a consideration of the possible
effects of substituents helps to account for the apparently random arrangement
of carcinogenic molecules within a chemically similar series.

A zo comipounds.

The literature on the azo compounds has been fully reviewed recently by
Badger and Lewis (1952). Although they discussed the various hypotheses
which have been advanced to account for the carcinogenicity of azo compounds
in terms of metabolism and in vitro reactivities, they were unable to come to
any conclusions. To the writer it seems that the azo compounds fall into four
classes: (i) the butter yellow group (4-dimethylaminoazobenzenes); (ii) amino-
azo compounds (like ortho-aminoazotoluene); (iii) compounds containing an
azo-2-phenol group and (iv) other azo compounds without an amino group.
The second and third of these classes are of special interest in regard to their
possible conversion to o-hydroxy amine derivatives.

Those azo dyestuffs which have been found to be carcinogenic experimentally
are oil soluble, hydrophobic compounds. From this it appears to be concluded
that water soluble azo compounds containing sulphonic and carboxylic acid
groups are eliminated from the body before they have an opportunity to induce
t;umours. The negative results obtained in the biological testing of these com-
pounds, quoted by Hartwell (1951) supports the conclusion. Recent work by

CH3                 C(H3

NH.   OH                                        OH    NH

'N   N-                      _N Na

So,:iN SNa                   =-=== SO Nit      'O NN

(XX)

45

4) . B. (CLA YSON

Gillman, Gillman and Gilbert (1949) and by Simpson (1952) on trypain blue
(XX) in the rat suggests that the introduction of a sulphonic acid group inay
not invariablv render the substance harmless.

(i) Butter yellow and its analogues: these compounds, in which an N-methyl
group appears to be essential for carcinogenicity, induce tumours almost exclu-
sively in the liver of the rat. Thus they differ from the carcinogenic aromatic
amines not only in their chemical structure but also in their restricted biological
activity. Mueller and Miller (1951) have shown that a substance which produces
formaldehyde is formed by the action of rat liver homogenates on 4-methyl-
aminoazobenzene. Hendry, Homer, Rose and Walpole (1951) suggest that this
substance may be 4-hydroxylmethylaminoazobenzene (XXI) which is analogous
to the cytotoxic methanolamines.

N  N X = X /   \-NH.('H.,OH

(XXI)

rFhe production in vivo of a cytotoxic agent could explain the cirrhosis and
subsequent, regeneration observed in the rat liver after administration of* butter'
yellow,' and its analogues. If it should be established that o-hydroxyamino-
derivatives are carcinogenic as a class it, will be necessary to determine whether-
the cytotoxic methanolamines or the hydroxylated metabolites are responsible
for- the initiation of the butter-yellow tumiours.

(ii) Amninoalzo conipounds: o-aminoazotoluene is the outstanding example
of this class. Crabtree (1949) examined this compound and-five of its isomers
for carcinogenic activity in rats and mice. He found, that whereas o-aminoazo-
toluene was carcinogenic in both rats and miciie, _2 :4'-dimethyl-4-aminoazobenzene
and 2': 5-dimethyl-2-aminoazobenzene were only carcinogenic to the mouse
liver. Crabtree (1 949) considered that the :3: 2'- and 4' : 3-dimethyl-4-amino-
azobenzenes were also carcinogenic in the mouse, but the evidence on -which
this contention is based is very meagre as only one mouse in each case showed
microscopic hepatomas. It is now suggested that as the active compounds are
para- substituted anilines their mode of action miay be explained by the hypo-
thiesis under discussion.

(iii) Azo-2-phenols: azo comipounds are often miade by coupling a phenol
an-d a, diazoniumi compound andi on occasion miay lead to an azo-2-phenol:

N   _N.6H5

e (r. I~~~~~~~~~~~~~ H

e 6H5N 2C1  -                                        NaCI.

Benzene-azo-2-napithol (PIeacock, 1948) is not only kiown to induce tumours in
mice but also would be expected to be nietabolised to I-amino-2-naphthol.
There is evidence to suggest that the latter is carcinogenic (Bonser and Jull,

466

MODE OF CARCINOGENESIS OF AROMATIC AMINES

personal communication). Previously, other similar azo compounds have been
tested and found inactive (Badger and Lewis, 1952). If the ideas expressed
here are valid it should be possible to find other carcinogenic azo- compounds
capable of reduction to the suspected carcinogenic ortho-hydroxy amines.

(iv) 2: 2'-Azonaphthalene and 2: 3'-azotoluene are examples of other azo
compounds which have been found to be carcinogenic but do not fit into any of
the above categories. It is not possible to suggest the nature of their mode of
action but the author was unable to detect any 2-amino-1-naphthol derivatives
in the urine of dogs fed with 2: 2'-azonaphthalene (unpublished observation).

The tendency, hitherto, has been to regard the azo group as the part of the
molecule conferring carcinogenicity. It is now suggested that this group is
only of importance insofar as it helps to form a molecule of a suitable structure
to carry functional groups which are themselves responsible for the biological
activity. If this is so, the similarities in both chemical configuration, and in
the induction of distant tumours, by the azo compounds, aminostilbenes and
amino-biphenyls are more readily comprehensible.

There seems to be no convincing evidence that aromatic amines containing
only a single ring are carcinogenic and therefore the present hypothesis has been
put forward in such a manner as to apply to compounds containing two rings or
more. On the other hand Walpole et al. (1952) have applied similar ideas, without
any additional experimental evidence, to explain the carcinogenicity of methylated
aminobiphenyls and have suggested that single ring ortho-hydroxyamines may be
carcinogenic. Their reasons for so doing are as follows: firstly, they quote an
industrial report of tumours of the bladder amongst workpeople handling crude
ortho-toluidine; secondly, they suggest that Crabtree's (1949) distribution of
carcinogenicity amongst the aminoazotoluenes is best accounted for by assuming
metabolism of the active compounds to 2-amino-3-hydroxytoluene (XXII),
(they overlook the possibility of direct hydroxylation to give phenylazo substi-
tuted ortho-aminophenols ;) and thirdly, they suggest that acceptance of their
postulate would give a possible mechanism for the mode of carcinogenesis of
2: 3'-azotoluene. However, it should be pointed out that the main experimental
evidence for the carcinogenicity of o-toluidline (Hartwell, 1951) is based on un-
published experimental work. The lack of reliable information in regard to the
carcinogenic action of single ring compounds is due to inadequate testing and
until good experimental evidence is available an open mind must be kept on
this point.

CH3

N12
\   JOH

(XXII)

SUMMARY.

1. It has been suggested that aromatic amines are carcinogenic because of
their conversion to ortho-hydroxy amines. This conversion is facilitated if the
position para to the aromatic amino group is blocked to biological hydroxylation.

467

D. B. CLAYSON

_              _

. . . . . . .

0.

I I I   I  + 1  +  +  +
II+ I++I I 1+  +  +

+
+l+

I +  I  I  I+  I  I  ++  +  +I-
+l  I I  I  I I  I  Il+  +f  I

+

+

IIA-A-l*I+ + AI+  +  I
I1++1Ill  1 1?  +  ?

-   0

0
0)  0  .

4 * * .   .  .  *   *   0

0.4.

_ .0._,.  . .._

_ 0~~ M1    01 01 001X  G

I I

++
++

I I
I I
I I

00

. . .

i + I

I I I
II I

I I I

+++

I I I

C. F. .

Ci   .   .i*C

.00

+
I

Ic
0

++ I + I

I I  I  I  i  I  I
+++?+  + +I
sI .   I  I  +  I

1+1+   + + I

I+++   + ? +

0w? 4Z -

MO   *

.)  U.  .- *   4Q* _

SO0 j  ''c

v

4;
00
4.

r

0

0

ca

, .

>  (D

1-4

00

0
0

O

, e

Q.

0

0

0

cc

ct

00

0.)
Co

;:
?Q
E.)

468S

- -      - - le It

?l      1?      -!Z? rzb  -t2 -t, ?

MODE OF CARCINOGENESIS OF AROMATIC AMINES

-~._

0

4Q0

0

+ I  I  I  0

+IS       1

T$       04~~~

I  II  I  *

-4Q~~

I II  I  o

0-

0

._

+III Ir

+11~ I

?11~~~~ ~1 11
f+    +  XCL

I II  +  toC

.  . 0 . .

0~ ~ !

Oo

... ..0

.0

0

0
0

0 0

U: N

-.
o o

.0

0 .

0 L

0 .

o O

4 C.)

~ 0

0D 0   -Z

0 )

0O

0

03

0

* 0E;

0

0 ;
0-1

*  H

m
0

EH

.4-
0

0..
CL

r..

(1)    1
4

4-'-)
0
42) 0

ce        (2) 4')

>      (L)  I

. - ca
4)       4Z    ;-4

P?q             4--)

-Q
0 0 .0 . 0 . 0 -~

.0
.   .   .   .   .   .

I   I1+   I  I   -,

i-4

I I I
I I I

I  I1+   I I  I  I  I

0 I
0 >

0

0c4

0   .0

+D 0  04  0  P4

4-

O6 -

g.

V _

Q. r

0

I-

1-

I11+1  1I 1 I1 1

0

0   0

0

0.40
I  I+ I  I  I  I  I  I  I  S

0++ 06  6   l 6   l  0

0  P-

a5   *^ . 0

1 CD  =

04   ad t

001 4--0

I   _

+m C .+ I 14  C' 4<

*- . -  v

.... 0.         . ..   .   .....,0O

000 m  X00    o 00  0
o ~ * ~ T h *  *   C L o  t

. GSo ~ 0   =    s I

. . . . . . .** -

0  0Smm_ m +C

469

c

D. B. CLAYSON

+ +~~~~~~~~~~~~~~~~~~~~~j

1 4

bo  bb~~

0~~~
0~~~

0)

-4  0~~

r4  Ce~~~0

0 T

-4

N~~~~

I 1+f

30

a)
I  I +  I +   I  I  I   0

14

>. ?

*4  1444  4 1

0>+ 00  0 0 0

04
a)

**    .  .   .

o        0no o

N

*; *g O

.0                 *               0

470

)0

14.  4-)   .4
a)   9      _

9 a) ( 4

4.

0

0
a)

14

4-11
r 4

0

+ +

I I
Og

a)t

> I I
;I

0l I

4-)
0.)

* .44

Ca)
*0!

0 s

I.

R

0

.

9
4-

0

10

.m C.

0
0

.D  .
0

0

z

*  a

0

4-) 0

a)

-   0

-o

_ z

4-) ;,

-   ,;

0 C

o a)

00
04

*0

* 0

0-4
Ca

01

I

MODE OF CARCINOGENESIS OF AROMATIC AMINES                  471
2. The similarities in chemical structure and biological activity of those
carcinogens which contain an aromatic amino group have been reviewed.

3. The need for further experimental work, both biological and biochemical,
has been emphasised.

REFERENCES.

ALLISON, J. B., WASE, A. W., LEATHEM, J. H., AND WAINIO, W. W.-(1950) Cancer

Res., 10, 266.

BADGER, G. M., AND LEwIs, G. E.-(1951) Brit. J. Cancer, 6, 270.
BAKER, K. W.-(1951) Acta Un. int. Cancr., 7, cif. 46.

BAKER, K. W.-(1952) IIme int. Congr. Biochim., Lons-le-Saunier (M. Declume),

p. 459.

BIELSCHOWSKY, F.-(1944) Brit. J. exp. Path., 25, 90.-(1945) Biochem. J., 39, 287.
Idem AND BIELSCHOWSKY, M.-(1952) Brit. J. Cancer, 6, 89.
BONSER, G. M.-(1943) J. Path. Bact., 55, 1.

Idem, CLAYSON, D. B., AND JULL, J. W.-(1951) Lancet, ii, 286.
Iidem, AND PYRAH, L. N.-(1952) Brit. J. Cancer, 6, 412.
Idem, AND GREEN, H. N.-(1950) J. Path. Bact., 62, 531.
CRABTREE, H. G.-(1949) Brit. J. Cancer, 3, 387.
ELSON, L. A.-(1952) Ibid., 6, 392.

GILLMAN, J., GILLMAN, T., AND GILBERT, C.-(1949) S. Afr. J. med. Sci., 14, 21.
HACKMANN, C. W.-(1951) Z. Kreb8forsch., 58, 56.

HADDOW, A., HARRIS, R. J. C., KON, G. A. R., AND ROE, E. M. F.-(1948) Phil. Tranm.,

241A, 147.

HARTWELL, J. L.-(1951) 'Survey of Compounds which have been Tested for Carcino-

genic Activity.' 2nd ed., Washington, D.C. (U.S. Publ. Hlth Serv.).

HENDRY, J. A., HOMER, R. F., ROSE, F. L., AND WALPOLE, A. L.-(1951) Brit. J.

Pharmacol., 6, 357.

HoCH-LIGETI, C.-(1947) Brit. J. Cancer, 1, 391.

HUEPER, W. C., AND WOLFE, H. D.-(1937) Amer. J. Path., 13, 656.
JULL, J. W.-(1951) Brit. J. Cancer, 5, 328.

MILLER, J. A., MILER, E. C., SANDIN, R. B., AND BROWN, R. K.-(1949) Cancer Res.,

9, 504.

Idem, MILLER, E. C., SANDIN, R. B., AND RusCH, H. P.-(1952) Ibid., 12, 283.

MORRIS, H. P., DUBNIK, C. S., AND JOHNSON, J. M.-(1950) J. nat. Cancer In8t., 10,

1205.

Idem, AND EYESTONE, W. H.-(1953) Ibid., 13, 1139.

MUELLER, G. C., AND MILLER, J. A.-(1951) Cancer Res., 11, 271.

NELSON, A. A., AND WOODARD, G.-(1953) J. nat. Cancer Inst., 13, 1497.
PEACOCK, P. R.-(1948) Ann. Rep. Brit. Emp. Cancer Campgn., 26, 198.

ROBINSON, D., SMiTH, J. N., AND WiLLiAMS, R. T.-(1951) Biochem. J., 50, 221, 228.
SIMPSON, C. L.-(1952) Brit. J. exp. Path., 33, 524.

SMITH, J. N., AND WILLIAMS, R. T.-(1949) Biochem. J., 44, 242.

SPITZ, S., MAGUIGAN, W. H., AND DOBRINER, K.-(1950) Cancer, 3, 789.

WALPOLE, A. L., WILLiAMS, M. H. C., AND ROBERTS, D. C.-(1952) Brit. J. industr.

Med., 9, 255.

WILLIAMS, R. T.-(1947) 'Detoxication Mechanisms.' London (Chapman Hall),

p. 138.

32

				


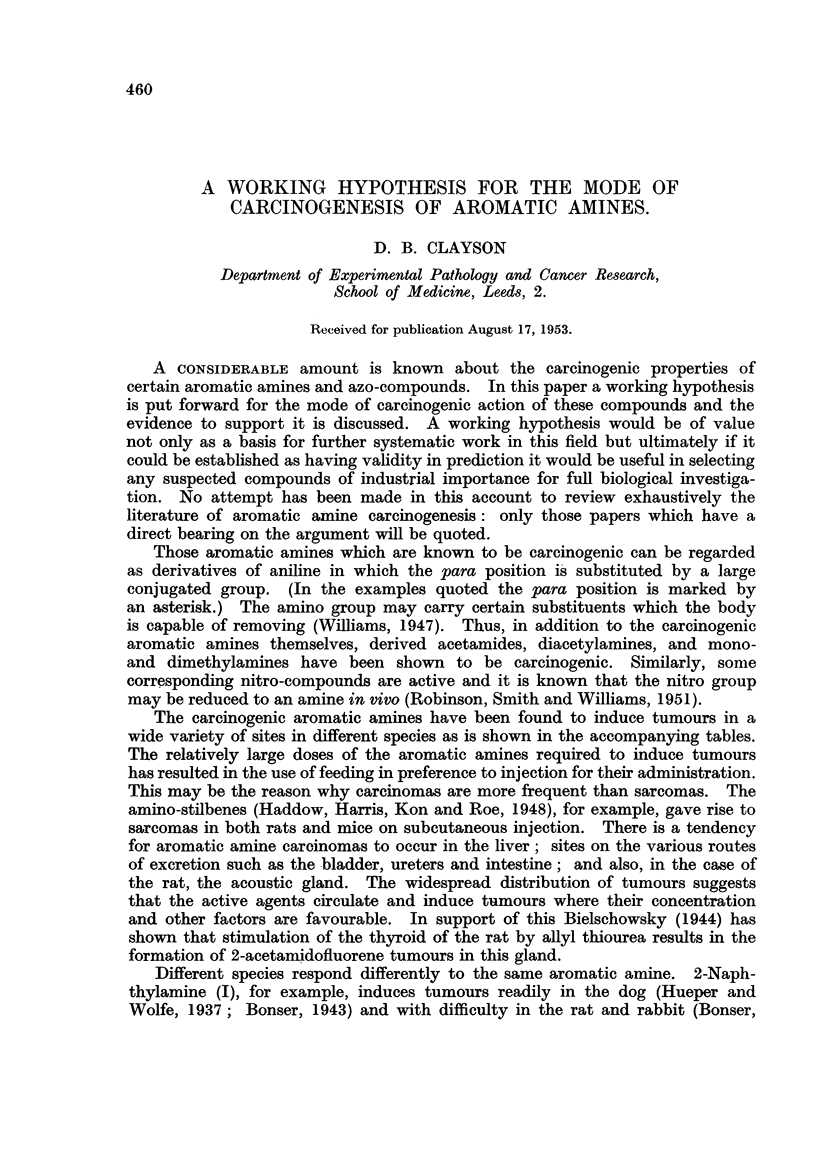

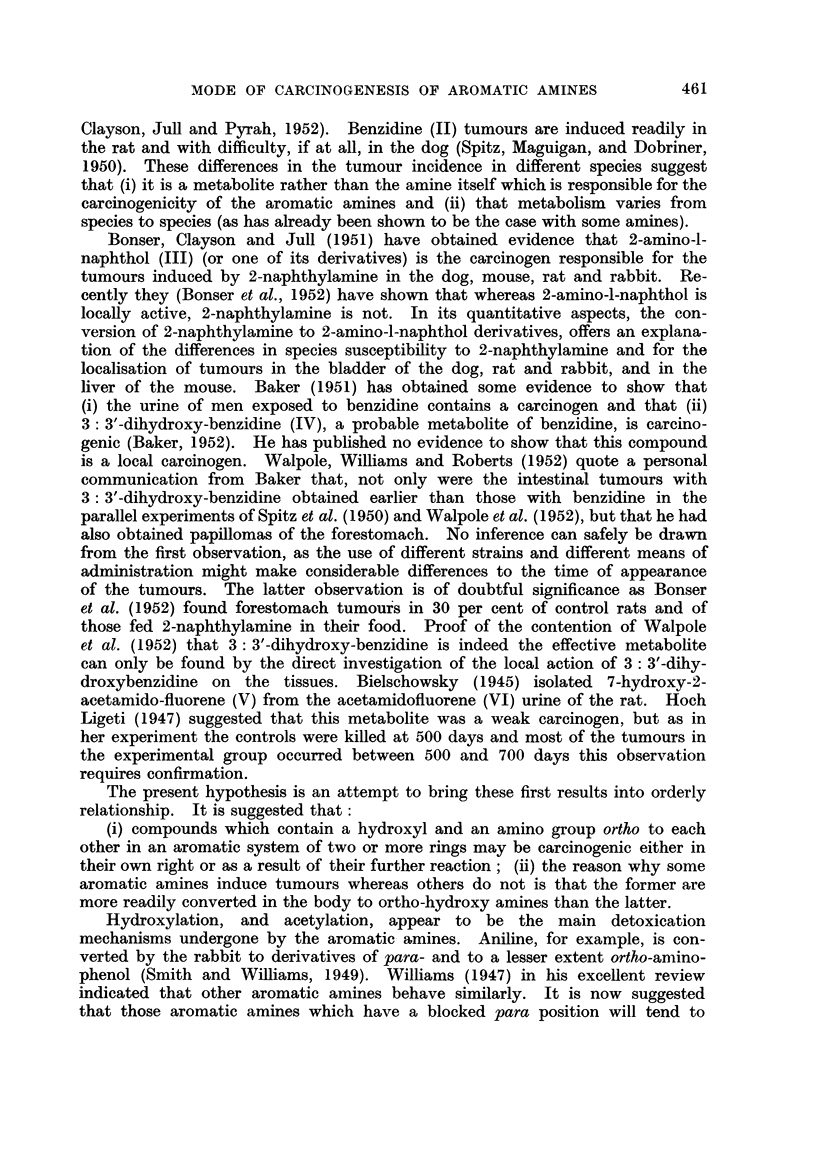

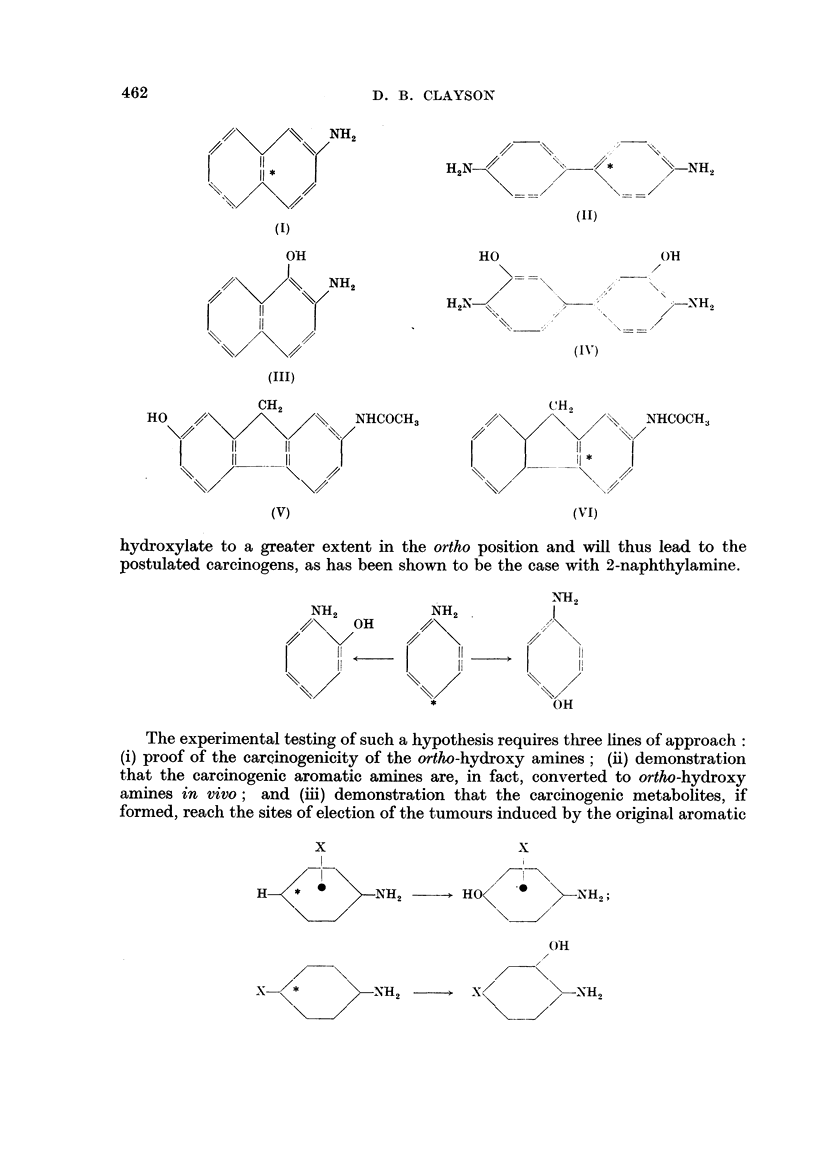

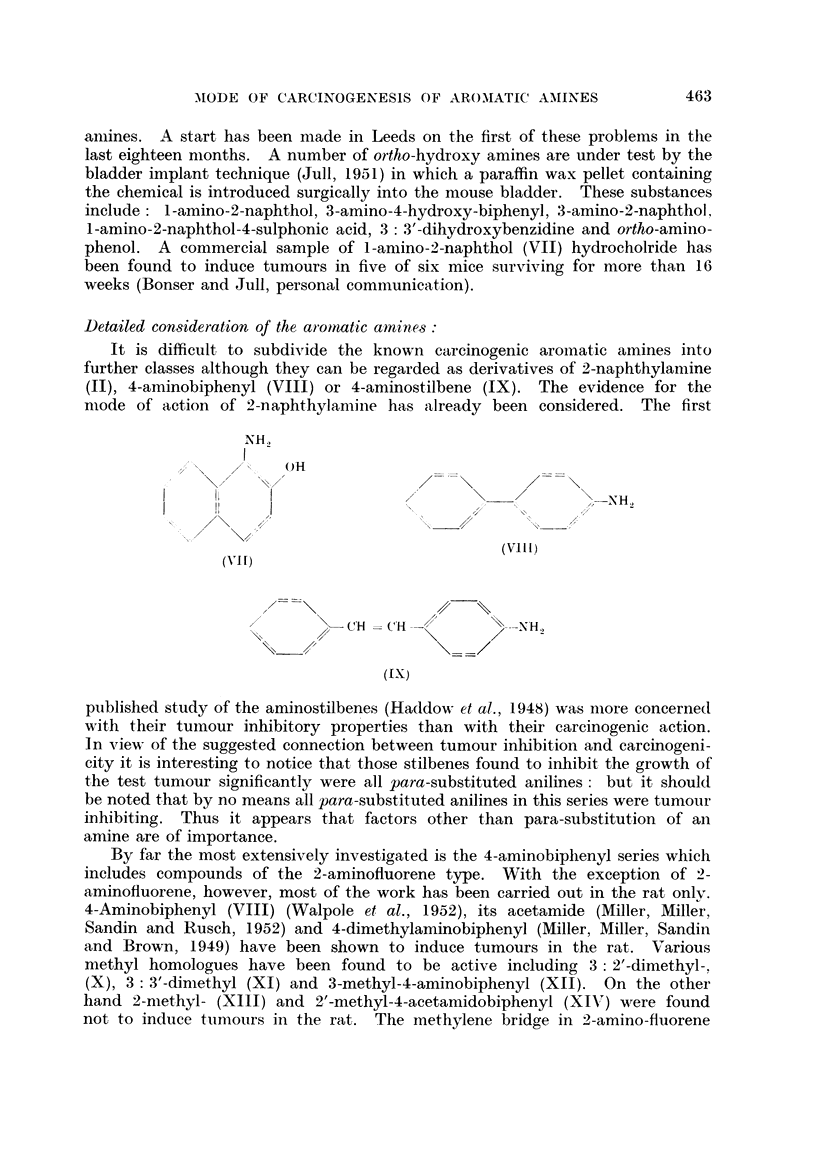

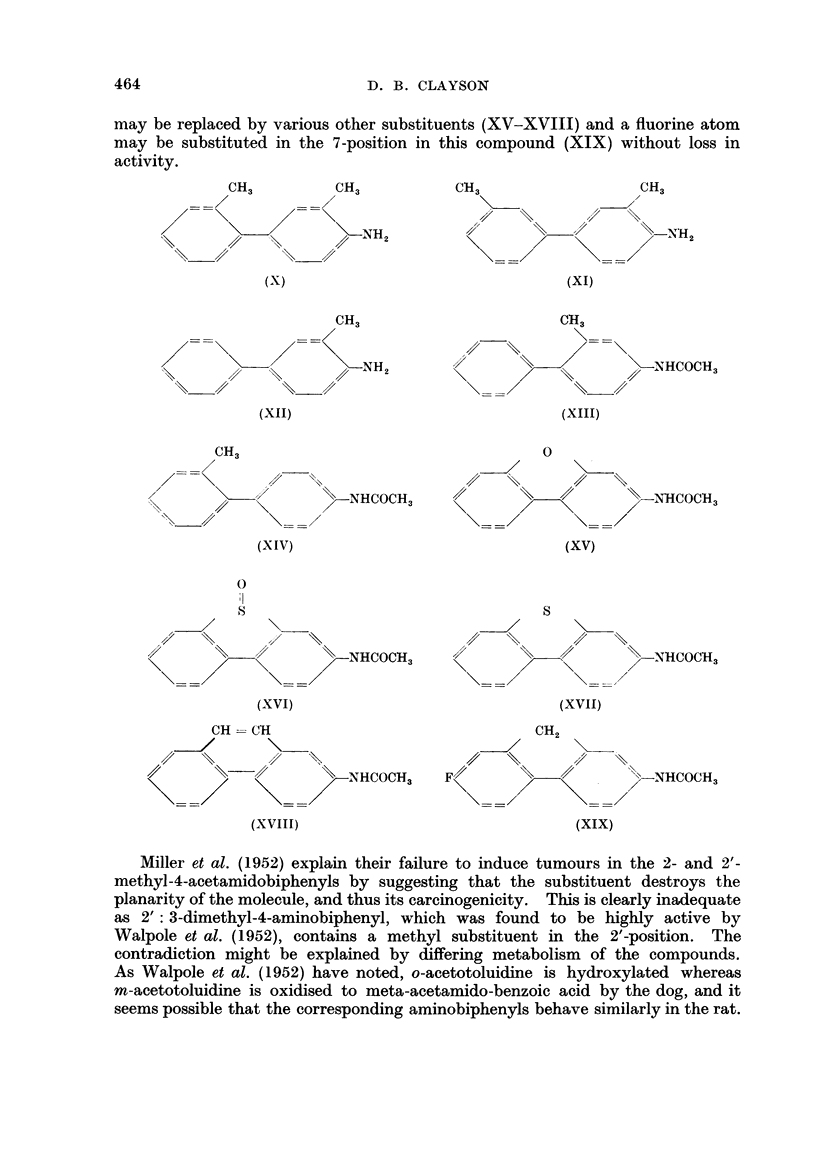

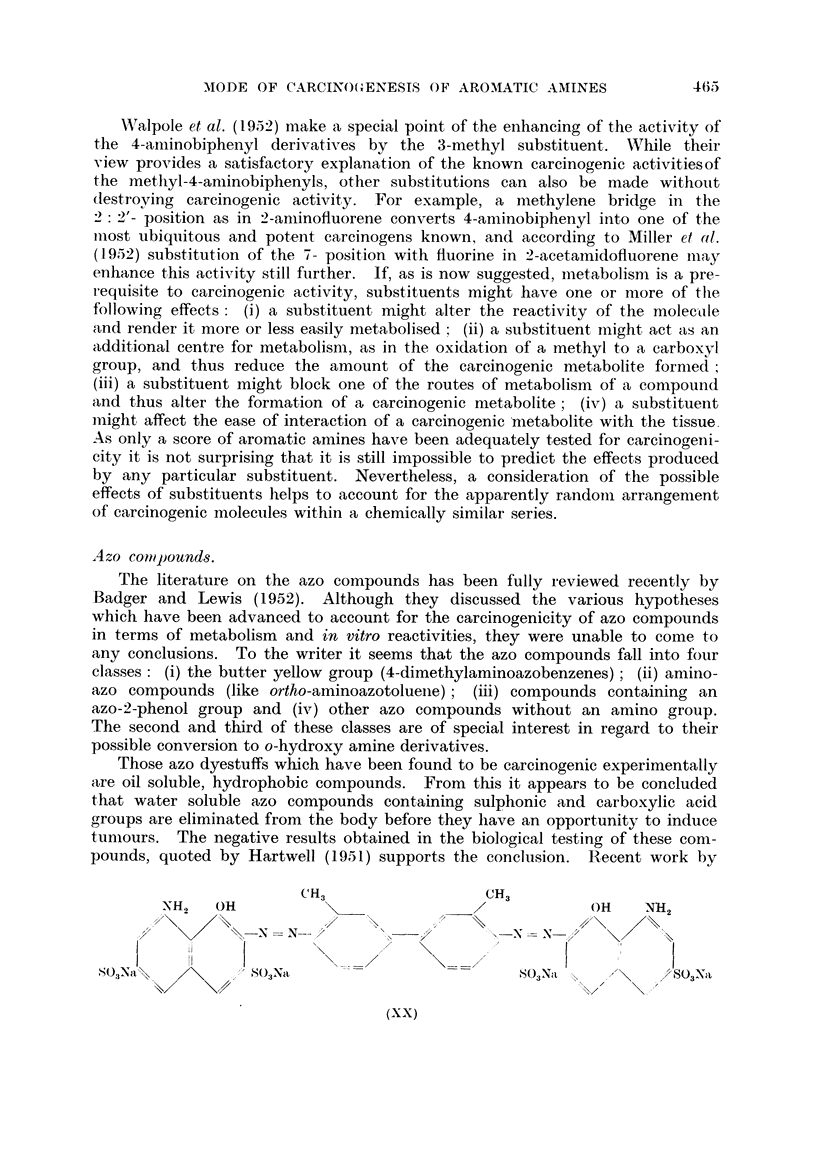

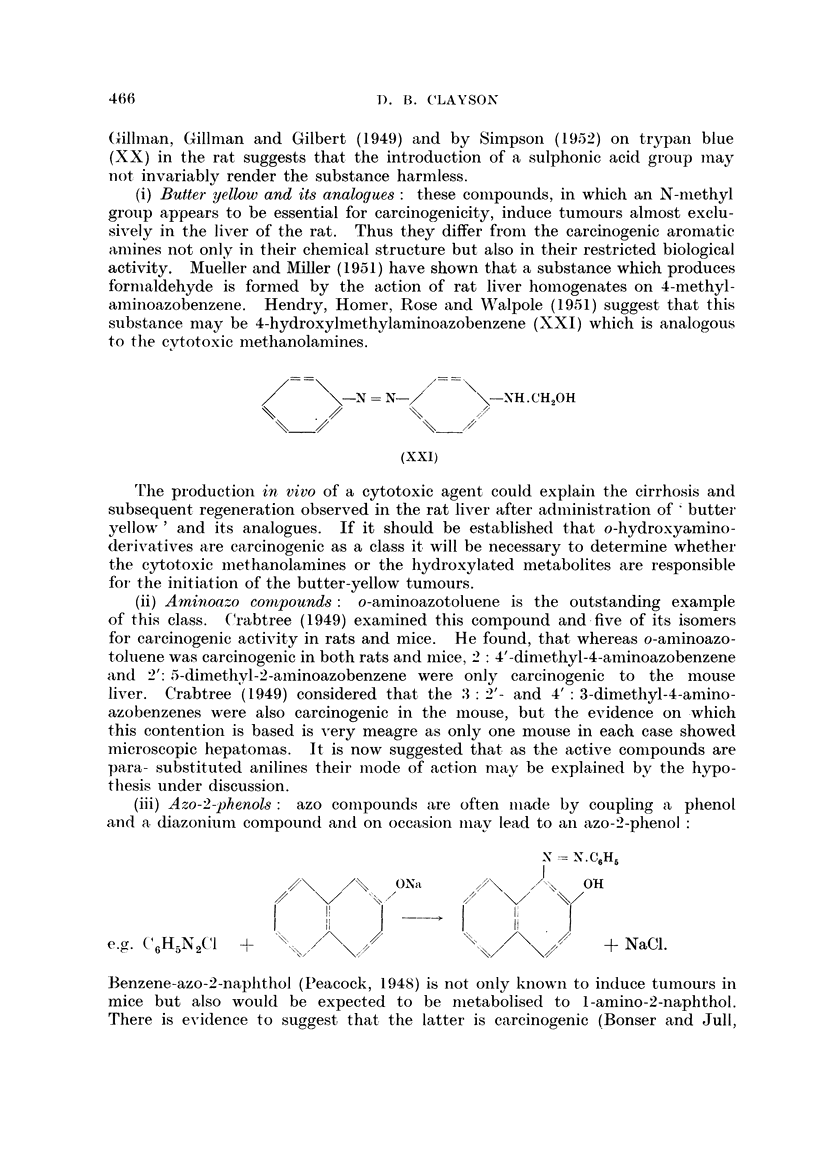

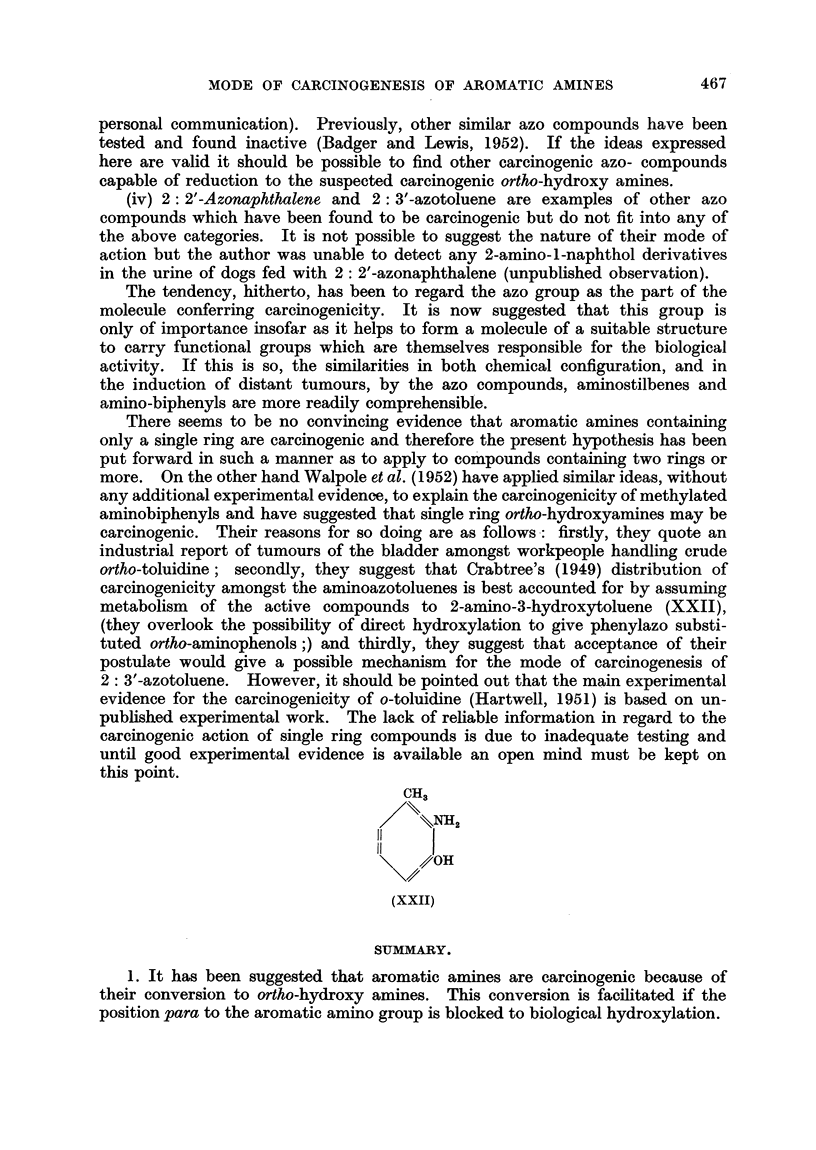

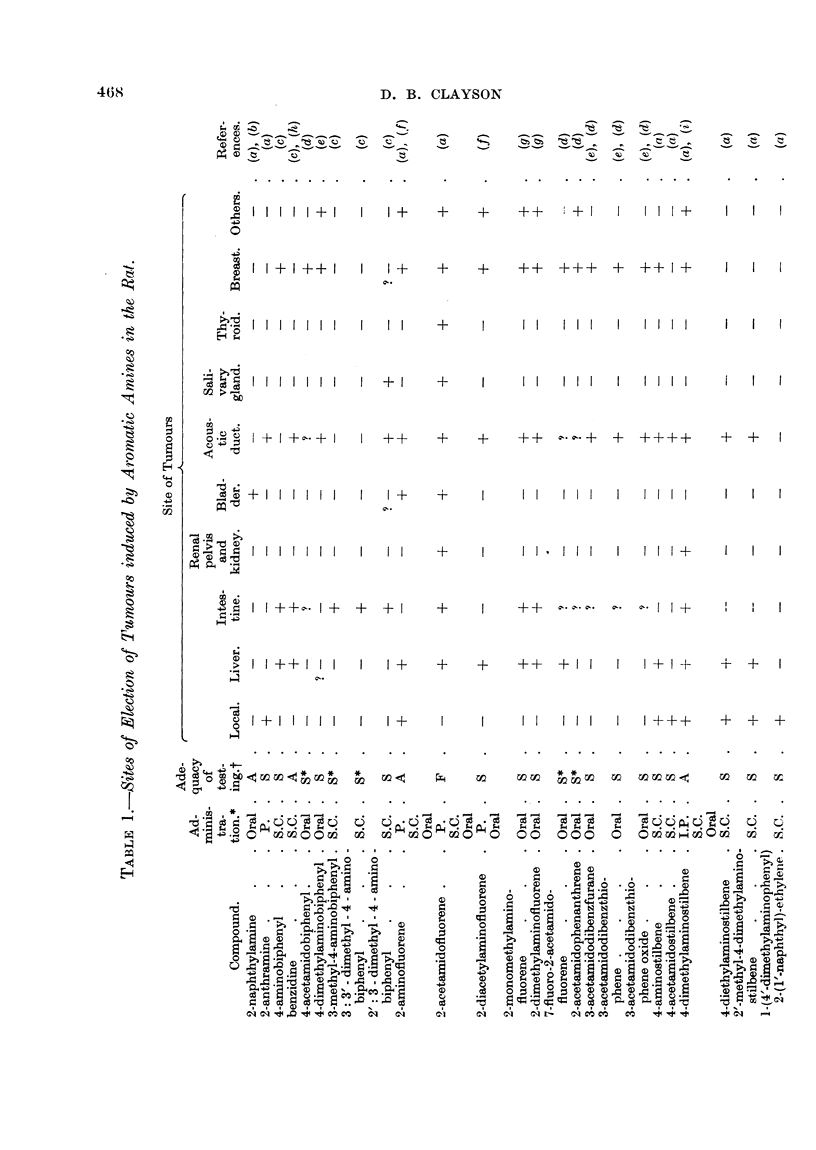

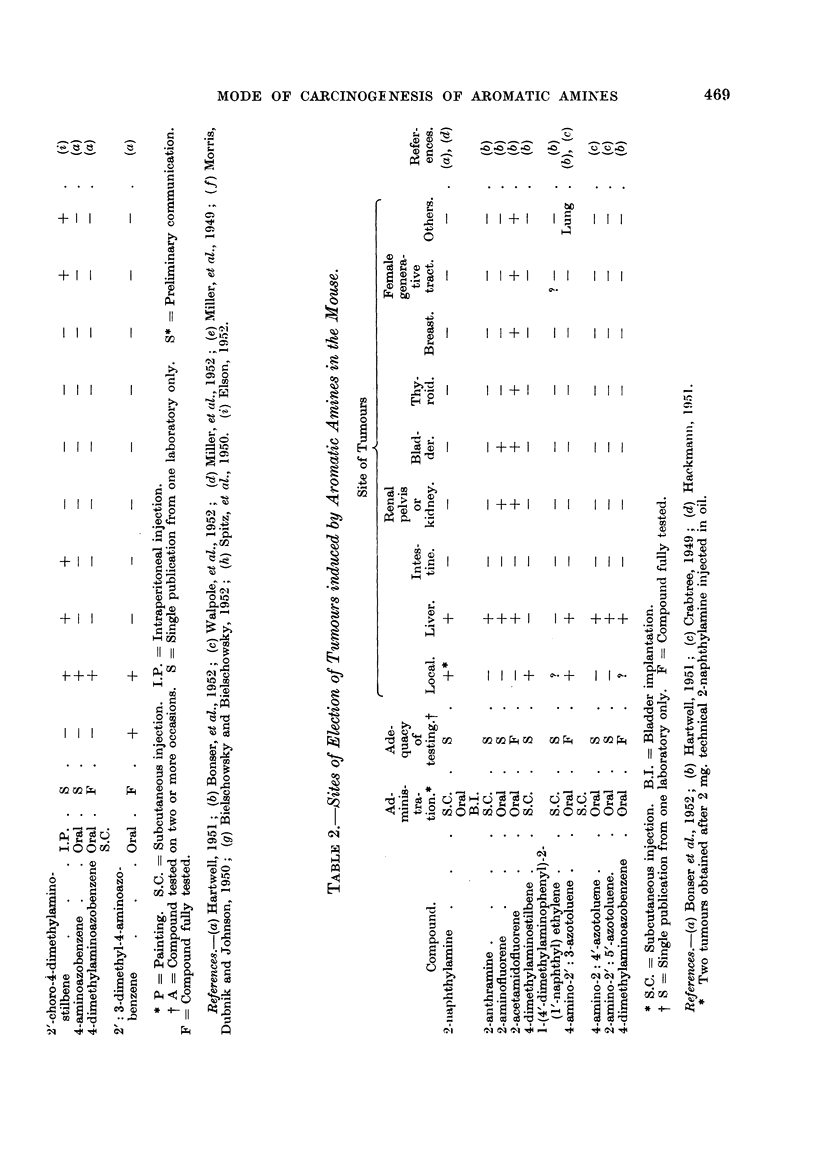

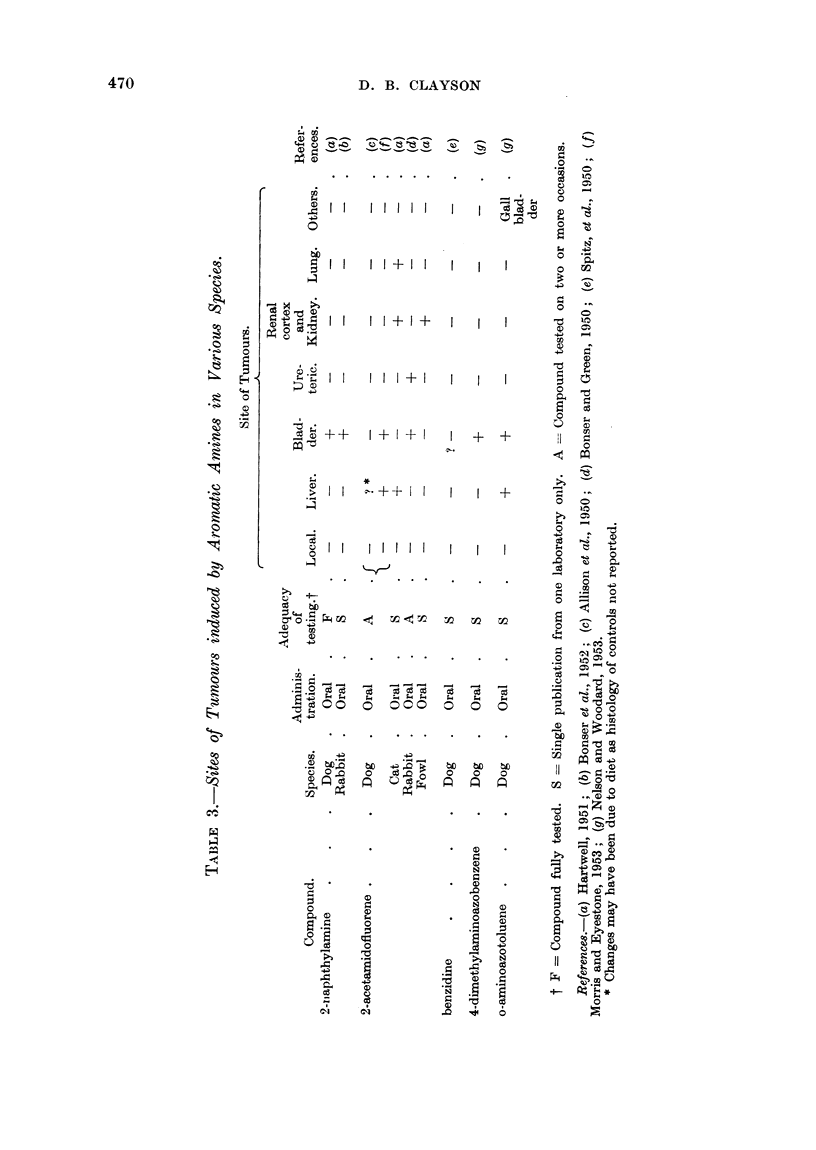

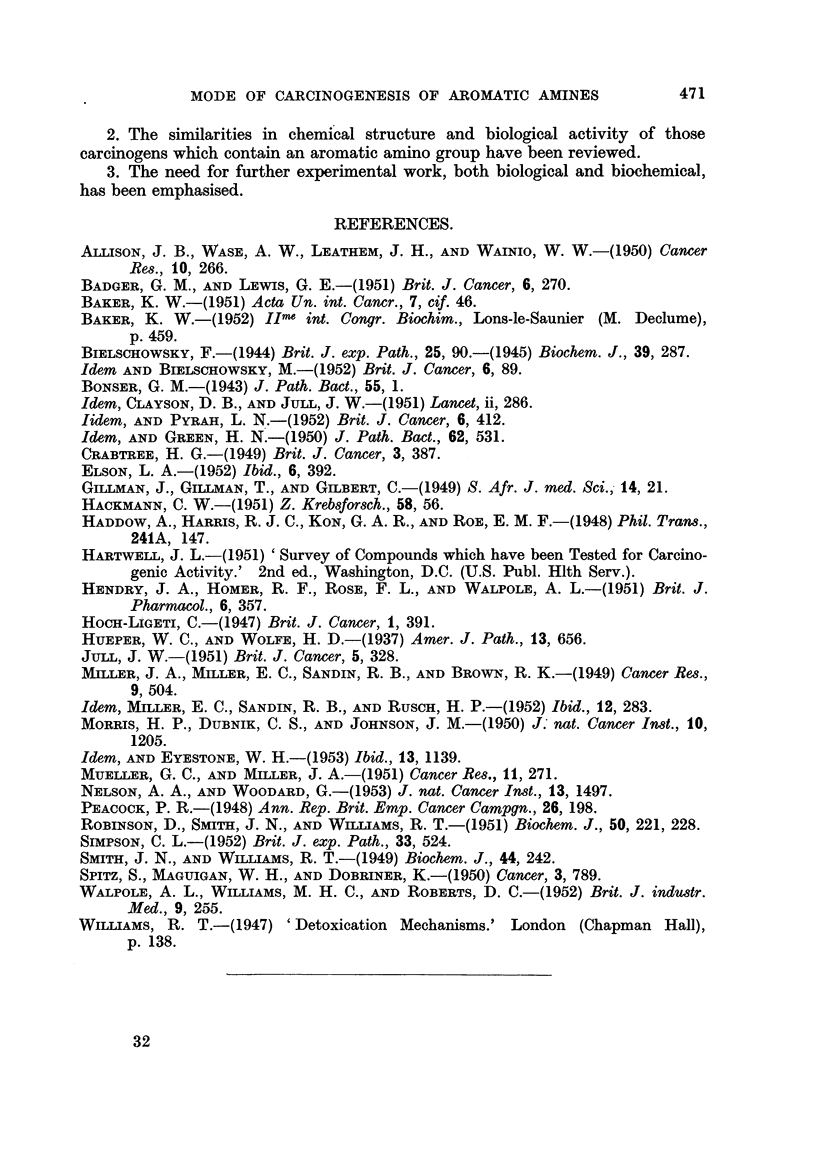

